# *Nannochloris* sp. JB17 as a Potential Microalga for Carbon Capture and Utilization Bio-Systems: Growth and Biochemical Composition Under High Bicarbonate Concentrations in Fresh and Sea Water

**DOI:** 10.3390/bioengineering11121301

**Published:** 2024-12-23

**Authors:** Giorgos Markou, Eleni Kougia, Dimitris Arapoglou

**Affiliations:** Institute of Technology of Agricultural Products, ELGO-Dimitra, Leof. Sofokli Venizelou 1, Lykovrysi, 14123 Athens, Greece

**Keywords:** bicarbonate, biochemical composition, carbon capture and utilization, biomass, microalgae cultivation

## Abstract

*Nannochloris* sp. JB17 has been identified as an interesting microalgal species that can tolerate high salinity and high bicarbonate concentrations. In this study, *Nannochloris* sp. JB17 was long-term adapted to increased bicarbonate concentrations (10–60 g NaHCO_3_ per L) in fresh or sea-water-based growing media. This study aimed to evaluate its growth performance and biochemical composition under different cultivation conditions. The highest biomass production (1.24–1.3 g/L) achieved in the study was obtained in fresh water media supplemented with 40 g/L and 60 g/L NaHCO_3_, respectively. Total protein content fluctuated at similar levels among the different treatments (32.4–38.5%), displaying good essential amino acids indices of 0.85–1.02, but with low in vitro protein digestibility (15–20%) rates. Total lipids did not show any significant alteration among the different NaHCO_3_ concentrations in both fresh and sea water (12.6–13.3%) but at increased sodium strength, a significant increase in unsaturated lipids and in particular a-linolenic acid (C18:3) and linoleic acid (C18:2) was observed. Carbohydrate content also ranged at very similar levels among the cultures (26–30.9%). The main fraction of carbohydrates was in the type of neutral sugars ranging from around 72% to 80% (of total carbohydrates), while uronic acids were in negligible amounts. Moreover, *Nannochloris* sp. showed that it contained around 8–9% sulfated polysaccharides. Since the microalgae display good growth patterns at high bicarbonate concentrations, they could be a potential species for microalgal-based carbon capture and utilization systems.

## 1. Introduction

The increasing concentration of anthropogenic greenhouse gas emissions (GHG), in particular, CO_2_ in the atmosphere is triggering a global ecological emergency, imposing actions for reductions in GHG emissions. Among the potential technologies, microalgae have been considered a promising biological technique for CO_2_ capture and utilization (bio-CCU) [1]. Microalgae are photosynthetic microorganisms that have gained much interest due to their ability to photosynthetically assimilate CO_2_ and to synthesize a broad range of high value-added products (proteins, lipids, pigments, etc.), which might be commercialized in the food or chemical industries [1,2].

Most commonly, CO_2_ is applied by sparging (diffusion) of gaseous CO_2_ into the microalgal growth medium. This method, however, has several limitations related to low dissolution efficiency and high outgassing rates where a considerable part of the undissolved CO_2_ is lost in the atmosphere [3,4]. To address this limitation, various microalgal bioreactor configurations or more efficient CO_2_ diffusing techniques have been developed and proposed [5]. However, each one has its drawbacks and cannot be applied easily in commercial-scale installations [5]. A potential alternative approach for utilizing CO_2_ for microalgae cultivation is to dissolve it first and convert it into bicarbonate ions (HCO_3_^-^). The dissolution of CO_2_ could be achieved in an alkaline absorption column (for example in NaOH solution), where CO_2_ is dissolved at significantly higher rates than in neutral media, minimizing its losses [6]. Bicarbonate thus might be an advantageous carbon source due to its potential lower supply costs and the more convenient photobioreactor design and development [6].

Many microalgal species are sensitive to relatively moderate-to-high bicarbonate levels. Concentrations (as NaHCO_3_) higher than 5 g/L negatively impact their growth [6,7]. Therefore, there is a need to screen, isolate, or adapt microalgal strains that can thrive under higher alkalinity and bicarbonate concentrations to achieve better utilization of CO_2_. So far, only a few attempts have been made to screen and isolate microalgal strains that demonstrate tolerance to relatively high bicarbonate concentrations [7,8,9,10,11,12,13]. *Nannochloris* sp. BJ17 has been identified as an interesting species due to its high tolerance to high bicarbonates and high salinity [12,14,15]. Previous studies on *Nannochloris* sp. BJ17 focused on the molecular mechanisms that define its bicarbonate and salt tolerance, however, there is limited information on assessing this species in regard to its biochemical composition and its potential to be used as a source of biomass for food or feed applications. Thus, in this work, we report the growth and biochemical composition of the green microalgal species *Nannochloris* sp. JB17 subjected to increased NaHCO_3_ concentrations in fresh and sea-water-based growing media after a prolonged period of adaptation at the given cultivation conditions.

## 2. Materials and Methods

### 2.1. Microorganism and Cultivation Conditions

The microalgae used in this study was a strain of *Nannochloris* sp. which was isolated in the premises of the Institute of Technology of the Agricultural Products (Greece) and more specifically it was isolated from an *Arthrospira platensis* culture, where *Nannochloris* sp. dominated against *Arthrospira*. Since *Arthrospira* is thriving in high bicarbonate concentrations and alkalinity (in Zarrouk medium consisting of 16.8 g/L NaHCO_3_, 2.5 g/L NaNO_3_, 1 g/L K_2_SO_4_, 1 g/L NaCl, 0.04 g/L CaCl_2_, 0.08 g/L Na_2_EDTA, 0.2 g/L MgSO_4_·7H_2_O, 0.01 g/L FeSO_4_·7H_2_O, and 1 mL/L of the trace element stock solution (2.86 g/L H_3_BO_3_, 0.02 g/L (NH_4_)_6_Mo_7_O_24_, 1.8 g/L MnCl_2_·4H_2_O, 0.08 g/L Cu_2_SO_4_, and 0.22 g/L ZnSO_4_·7H_2_O), the occurrence and domination of *Nannochloris* sp. raised particular interest as species that could be cultivated in such high bicarbonate growing media. It is worth noting that high bicarbonate concentrations and high salinity could be a way to avoid biological contamination with other unwanted microalgae enhancing the commercial viability of the microalgae [16].

All cultures (stock and experimental) were performed in a controlled room with a steady temperature of 28 ± 2 °C in closed systems of glass photobioreactors (total volume 1 L, diameter 102 mm), whereas the cultivation was applied in a batch mode. As a light source, a tube fluorescent lamp of 57 Watt was used, with cool white light, placed on one side of the photobioreactors, while the photoperiod was set to 16/8 light-dark cycle, and light intensity was set to 150 μmol/m^2^/s (measured by SpectraPen, PSI, Drásov, Czech Republic). Agitation through continuous aeration at about 2 L air per L culture per minute with filtered (0.2 μm filter) atmospheric air was achieved using a membrane air pump (ACO-308, Guangdong Hailea Group Co. Ltd., Guangdong Province, China).

### 2.2. Isolation and Adaptation of Microalga

The microalga was isolated through the streak plate technique in Petri dishes with agar (1%) and Zarrouk medium as a nutrient source. After the conduction of two sequential streak plate isolations, the microalga was identified by DNA sequencing (see 2.3 Analytical Methods) as *Nannochloris* sp. JB17 (100% identity). *Nannochloris* sp. JB17 was then cultured for mass production in Zarrouk medium with 20 g/L NaHCO_3_. Next, the microalga was transferred in a modified Zarrouk medium under four different concentrations of NaHCO_3_ (10, 20, 40, and 60 g/L) in fresh as well as in artificial sea water (distilled water containing 33 g/L natural sea salt) (for more details see supplement material). The cultures were performed for a prolonged period of time (at least 14 weeks) to the corresponding bicarbonate concentrations for adaptation purposes before conducting the experiments. The adaptation of the microalgae to the different conditions was chosen as a strategy to avoid the stress effects resulting from any sudden change of the bicarbonate and salinity. For the adaptation of the microalga, the cultures were performed in batches applying renewals (20% inoculums in freshly prepared media) every 2 weeks. Since the double time of cells was approximately 2 days, the total amount of generation throughout the adaptation period was estimated to be more than 45.

Throughout the text, the cultures were abbreviated as N_10_, N_20_, N_40_, and N_60_ for the experimental series containing 10, 20, 40, and 60 g/L NaHCO_3_, respectively, and SW_10_, SW_20_, SW_40_, and SW_60_ for the experimental series containing 10, 20, 40, and 60 g/L NaHCO_3_ and 33 g/L natural sea salt, respectively. Regarding the sodium ion Na^+^, the cultures N_10_, N_20_, N_40_, and N_60_ contained 3.8, 6.5, 12, and 17.5 g-Na^+^/L, whereas the cultures in sea water SW_10_, SW_20_, SW_40_, and SW_60_ contained 16.4, 19.1, 24.6, and 30.1 g-Na^+^/L, respectively. All cultures were performed in triplicates and their mean (±standard deviation) were given.

### 2.3. Analytical Methods

The daily growth of *Nannochloris* sp. was determined spectrophotometrically by measuring optical density (OD) at 750 nm. Biomass dry weight was directly measured at the end of the cultivation period, after repeated cycles of washing the biomass with distilled water and its subsequent drying at 105 °C until constant weight. All further analyses were performed afterward on lyophilized biomass through a Freeze Dry System (Thermo Savant, ModulyoD). Total proteins, carbohydrates, and lipids were assayed according to the methods of Lowry, et al. [17], Kochert [18], and Mishra, et al. [19], respectively, which are described in more detail in previous work [20]. Chlorophyll and total carotenoids (mg/L) were extracted with 90:10 methanol to deionized water ratio (MeOH:DW) at 70 °C for 10 min and calculated by the formulas given by Lichtenthaler [21]. Ash content was determined through biomass incineration (overnight) at 550 °C.

The fatty acid profile was determined through Gas Chromatography (Shimadzu Nexis GC-2030 equipped with a Dielectric Barrier Discharge Ionization Detector). Regarding the chromatography conditions, a capillary column Mega-10 (0.25 mm/0.25 μm, 60 m) was used. The temperature was set to 60 °C with a step of 2 °C/min, while split temperature and detector temperature were set to 245 °C. Lipids were initially extracted from 10 mg biomass using 1.5 mL of CHCl_3_: MeOH at a ratio of 2:1, then 0.2 mL of saturated water for phase separation was added and evaporation of the organic phase in nitrogen gas was followed by the addition of 1 mL hexane. Esterification of lipids was performed through the addition of 50 μL KOH. The method was calibrated by a solution of FAMEs (Supelco 37 Component FAME Mix).

For the determination of total phenolics and antioxidants, extraction was initially performed according to Goiris, et al. [22] using EtOH:DW (3:1) as a solvent. Total phenolic content was assayed afterward, through a slightly modified method referred to in Hajimahmoodi, et al. [23]. Briefly, 0.1 mL of a sample containing the extracted phenols was mixed with 0.75 mL of 10% Follin-Cioclteu reagent. After 5 min, 0.75 mL of 7.5% sodium carbonate was added to the mixture, and OD was recorded after 90 min at 750 nm. The results were expressed as gallic acid equivalent (GAE)/g dry biomass. The antioxidant capacity of the extracts was determined according to a slightly modified method of ABTS·^+^ (2,2’-azino-bis(3-ethylbenzothiazoline-6-sulfonic acid) method referred to in Li, et al. [24]. Shortly, a radical cation was generated through the reaction of 2.45 mM potassium persulfate with 7 mM ABTS for 16 h in the dark and the resulting solution was diluted with EtOH in order to give an absorbance of 0.7 at 734 nm. Afterward, 2 mL of the ABTS·^+^ solution was mixed with 0.1 mL extract and after 6 min, the OD was measured at 734 nm, while the results were expressed as Trolox equivalent (TEAC)/g organic matter.

In vitro protein digestibility was assayed according to the method referred to in Almeida, et al. [25]. Specifically, in 25 mg of dry biomass, 0.25 mL of 0.1 M HCL containing 1.5 mg/mL pepsin was added, and subsequently, the samples were incubated for 3 h at 37 °C in order for the hydrolysis to take place. Next, 0.75 mL of 0.5 M NaOH was added to the mixture and pancreatic digestion was followed with the addition of 1 mL (0.2 M, pH 8) phosphate buffer containing 10 mg of enzyme pancreatin. Subsequently, 0.1 mL of 0.005 M sodium azide was added (in order to prevent microbial growth), and the samples were incubated at 37 °C overnight, after which 0.1 mL of 10% TCA was added. The supernatant was collected after centrifugation (503× *g* for 20 min) and was subjected to analysis according to the Lowry method [17] while free amino acids were determined according to the EBC-ninhydrin method [26]. Amino acids were analyzed through High-Performance Liquid Chromatography (HPLC) after biomass hydrolysis with HCl at 120 °C for 24 h. The employed HPLC system (Shimadzu, HPLC Nexera X3 RF-20AXS, Kyoto, Japan) was equipped with an RF-20AXS fluorescence detector and SIL-40C Auto Sampler. Pre-column sample derivatization was performed using 9-fluorenylmethyl chloroformate (FMOC-Cl), o-phthalaldehyde (OPA), and 3-mercaptopropionic acid, all purchased by Merck (Sigma Aldrich). Mobile phase A contained 10 mM Na_2_HPO_4_, 10 mM Na_2_B_4_O_7_, pH 8.2, and 5 mM NaN_3_. Mobile phase B contained acetonitrile:methanol: water (45:45:10, *v*:*v*:*v*). The method was calibrated by a solution of 17 amino acids standards (aspartic acid, glutamic acid, serine, histidine, glycine, threonine, arginine, alanine, tyrosine, cysteine, valine, methionine, phenylalanine, isoleucine, leucine, lysine, proline; Supelco AAS18 Amino Acid Standard). For the chromatography, an Agilent ZORBAX Eclipse Plus C18 column was used. The flow rate of eluents was 1.5 mL/min and the column temperature was 40 °C. The essential AAs index was calculated based on Oser [27] using whole egg protein as the reference.

Colorimetric determination of neutral sugars was performed according to a resorcinol sulfuric acid method [28], while quantitative analysis of uronic acids was also performed according to the method referred by [29]. Sulfated polysaccharide content was estimated after the extraction of polysaccharide sulfate with the addition of 2 M HCl and the subsequent placing of the samples at 120 °C overnight [30]. Polysaccharide sulfate content was then determined with a turbidimetric assay [31]. Briefly, 0.1 mL of the hydrolysate was mixed with 1.9 mL of 3% TCA and 0.5 mL of BaCl_2_-gelatin, and OD was recorded after 20 min at 360 nm.

Chlorophyll-a fluorescence analysis (maximum photochemical yield of PSII and non-photochemical quenching) was performed with the handheld PAM fluorometer AquaPen (Photon Systems Instruments, Czech Republic) following internal analysis protocols. Chlorophyll-a fluorescence was performed in dark (20 min) adapted cells [32].

The microalgal genus was identified by sequencing using primer pairs for Eukaryotes. DNA was extracted (from ~3 μg lyophilized biomass) using a commercial DNA extraction kit (MACHEREY-NAGEL, Düren, Germany) and was sequenced after Illumina miSeq using primers specific for Eukaryotes. The sequences were detected, grouped in Operational Taxonomic Units (OTUs) using a 97% identity threshold and identified using the BLASTN algorithm of SILVA database (https://www.arb-silva.de/) (accessed 25 April 2022) by Smallomics. Data analysis and visualization were performed using the package Phyloseq v. 3.6.1 (Rstudio (2021.09.2+382)) [33].

Where relevant, statistical analysis was based on one-, and/or two-way analysis of variance (ANOVA), performed using SigmaPlot 12.0 software (Systat Software, Inc., San Jose, CA, USA). All data were tested for Normality (Shapiro–Wilk test) and for equal variance between treatments. Post-hoc statistical analysis was based on Duncan’s pairwise multiple comparison procedure.

## 3. Results and Discussion

### 3.1. Biomass Growth and Photosynthetic Performance

**Figure 1:**
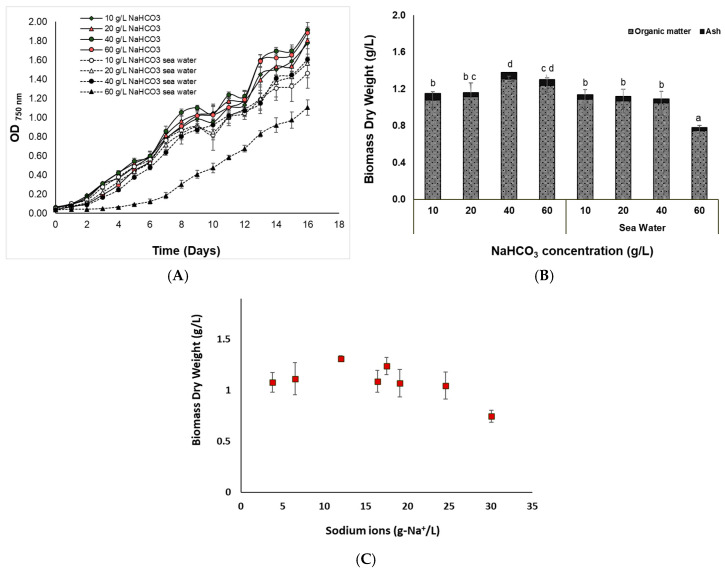
(**A**) Biomass growth kinetics, (**B**) dry weight of *Nannochloris* sp. JB17 cultivated for 16 days in different NaHCO_3_ concentrations in fresh as well as in sea water, and (**C**) biomass dry weight of *Nannochloris* sp. in relation to the total sodium ions (Na^+^). The data represent the mean of *n* = 3, while error bars represent the standard deviation. Different subscript letters denote statistically significant differences (*p* = 0.05).

In Figure 1, the growth kinetics (Figure 1A) of the isolated *Nannochloris* sp. for a cultivation period of 16 days after the given conditions and the final produced dry weight (Figure 1B) are illustrated. Among the different cultures, the best dry weight production was achieved in N_40_ and N_60_ (1.3 ± 0.03 and 1.24 ± 0.08 g/L; *p* > 0.05), while the lowest growth performance and value of dry weight (0.7 ± 0.06 g/L) was observed in SW_60_, while no differences were observed between N_10_, N_20_, SW_10_, SW_20_, and SW_40_. However, a two-way ANOVA analysis showed that there was a statistically significant difference between the cultures in fresh and sea water, meaning that the cultures in sea water had overall lower biomass production (*p* < 0.05). This was most probably due to the negative effect of sodium ions on the growth of the microalga (Figure 1C), especially at the highest sodium ion concentrations. These findings agree with those of Liu, et al. [31] where an isolated strain *Nannochloris* sp. SAE1, which was highly homologous to *Nannochloris* sp. BLD-15 had a slower growth rate in the cultures treated with high NaCl than with high NaHCO_3_ concentrations (up to 1 M, i.e., 84 g/L).

While NaHCO_3_ concentration at certain levels can promote the growth of microalgae, at elevated concentrations inhibition can occur. Although high concentrations of NaHCO_3_ are inhibiting for most microalgae, *Nannochloris* sp. BJ17 is particularly tolerant as was also demonstrated by other studies [12,14]. According to these studies, the viability of the cells and the final dry weight of *Nannochloris* sp. JB17 was higher with the addition of NaHCO_3_ concentration up to 25 g/L compared to control (4.2 g/L NaHCO_3_). In the study of Qiao, et al. [15], *Nannochloris* sp. was isolated from an extreme saline-alkali soil and was examined for its salt-tolerance abilities in a range of NaHCO_3_ and NaCl concentrations. Results revealed that *Nannochloris* sp. had an optimal growth at 42 g/L NaHCO_3_ and could survive up to 84 g/L, while it also could survive in the presence of 35 g/L NaCl and at the simultaneous presence of 17 g/L NaCl and 25 g/L NaHCO_3_, respectively.

**Figure 2:**
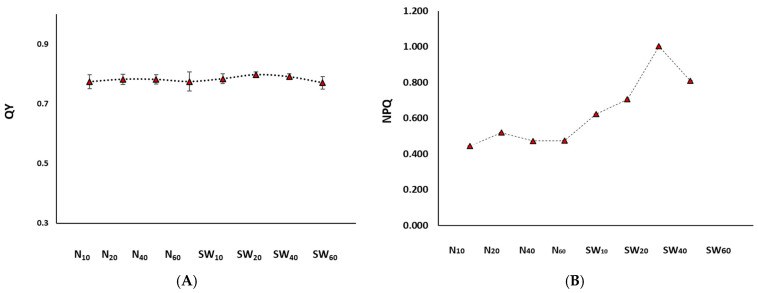
(**A**) Maximum photochemical yield of PSII (QY), and (**B**) non-photochemical quenching (NPQ) of *Nannochloris* sp. JB17 cultivated for 16 days in different NaHCO_3_ concentrations in fresh as well as in sea water. The data represent the mean of *n* = 3, while error bars represent the standard deviation.

Chlorophyll-a fluorescence analysis is a tool that allows for a closer examination of the photosynthetic efficiency of photosynthetic organisms subjected to varied environmental conditions [34]. As shown in Figure 2A, the maximum photochemical yield of PSII (quantum yield; QY) of dark-adapted cells ranged between 0.77 and 0.8. In healthy green microalgal cultures, the QY is around 0.8 and its decline represents a situation when microalgae become stressed [35]. The results obtained in this study suggest that none of the experimental cultures of the present study was stressed in a way that it could impair the photosynthetic machinery of the microalgae. However, the non-photochemical quenching (NPQ) analysis (Figure 2B) shows that the microalgae grown on sea water were less efficient for photochemistry [36] since they had higher NPQ values, which might explain somehow the lower biomass production. The NPQ values were positively correlated to the sodium ion (see Supplementary Matrials) with a relatively strong correlation efficiency (R^2^ = 0.65). The results suggest that even though the PSII yield (QY) was not impaired, at the increased sodium strength the cells might have a reduced electron flow at the acceptor side of PSII [36] leading to lower photochemistry upon the need for higher photoprotection actions [37]. Since the experiments were conducted after long-term adaptation to the given environments, the QY results could lead to the conclusion that the cells had reached homeostasis, so any effect on the growth was an outcome of optimal or suboptimal conditions rather than the result of any stress stimuli [38] caused by a sudden change of the environmental conditions (i.e., by the increase of NaHCO_3_ and NaCl concentrations).

Chlorophyll-a and chlorophyll-b showed a positive correlation with the increase of sodium ions (g-Na^+^/L) in the media. Specifically, for chlorophyll-a, the highest value was obtained in SW_60_ (30.1 g-Na^+^/L), while for chlorophyll-b, SW_20_ gave the highest values. A two-way ANOVA revealed that the chlorophyll (a and b) content was higher in the group of the cultures grown in salt water compared to the group of fresh water (*p* < 0.05). High content of chlorophyll which is an essential component of the photosynthetic apparatus, is related to higher photosynthetic activity in microalgae or higher plants [12,39], which is in line with the unvaried QY values obtained in this study. According to Wang, et al. [12], NaHCO_3_ in the culture of *Nannochloris* sp. JB17 promotes the synthesis of chlorophyll-a since it gave up to 1.5-fold higher chlorophyll-a content compared to control (4.2 g/L NaHCO_3_). It was found that numerous genes within *Nannochloris* sp. JB17 cells operating in various facets of photosynthesis exhibit a response to NaHCO_3_. This implies that *Nannochloris* sp. JB17 might enhance their photosynthetic efficiency as an adaptive mechanism to counteract the high NaHCO_3_ concentrations [14].

**Figure 3:**
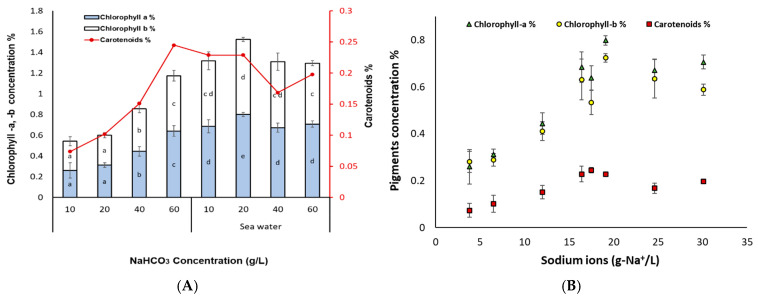
(**A**) Pigments concentration of *Nannochloris* sp. JB17 for the cultures treated with different NaHCO_3_ concentrations in fresh as well as in sea water, and (**B**) pigments concentration in relation to the total sodium ions (Na^+^). The data represent the mean of *n* = 3, while error bars represent the standard deviation.

**Figure 4:**
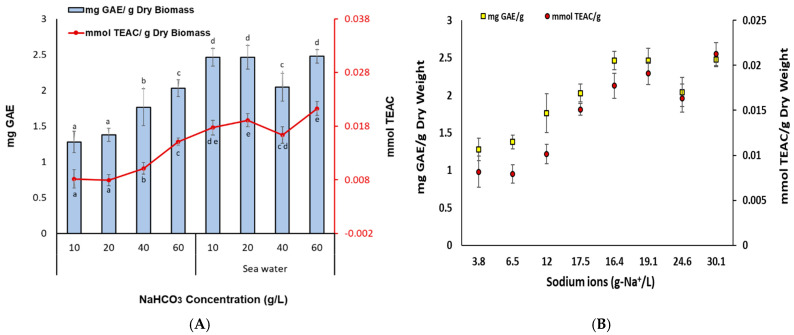
(**A**) Total phenolic content and antioxidant capacity of *Nannochloris* sp. JB17 for the cultures treated with different NaHCO_3_ concentrations in fresh as well as in sea water, and (**B**) in relation to the total sodium ions (Na^+^). The data represent the mean of *n* = 3, while error bars represent the standard deviation. Different subscript letters denote statistically significant differences (*p* = 0.05).

As regards the total carotenoids (Figure 3), there was also a positive correlation with the increase of sodium ions up to the concentration of N_60_ (17.5 g-Na^+^/L highest value), while a further increase led to a slight decrease (SW_40_ and SW_60_). Carotenoids act as photoreceptors in the photosystems and being good antioxidants, they display protective effects against photodamage by quenching active oxygen species or triplet excited state chlorophyll [40,41]. The increased content of carotenoids comes in line with the higher NPQ values observed in the cultures grown with increased sodium ion concentrations. Moreover, the total antioxidant capacity (Figure 4) followed also the same trend with the highest value arising again for treatment SW_60_ (0.02 mmol TEAC/g). As it seems, the total antioxidant capacity increased with higher NaHCO_3_ and sodium ion concentrations.

According to Wang, et al. [12], both intracellular MDA (malondialdehyde) levels and antioxidant enzyme activity showed an increase in response to higher NaHCO_3_ concentrations and prolonged exposure durations. Lipid peroxidation was notably elevated in treatments with 25 g/L NaHCO_3_ and longer durations of treatment. Importantly, heightened activity levels of peroxidase (POD), catalase (CAT), and superoxide dismutase (SOD) suggested that *Nannochloris* sp. mitigated oxidative injury by enhancing their ability to neutralize reactive oxygen species (ROS) induced by exposure to NaHCO_3_.

### 3.2. Biochemical Composition

**Figure 5:**
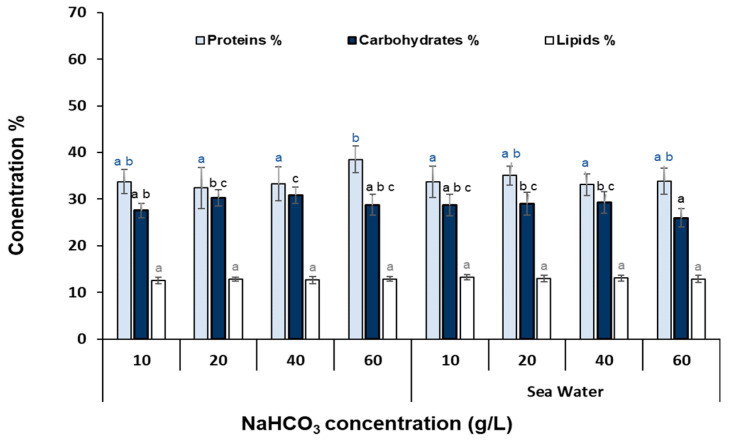
Biochemical composition of *Nannochloris* sp. for the cultures treated with different NaHCO_3_ concentrations in fresh as well as in sea water. The data represent the mean of *n* = 3, while error bars represent the standard deviation. Different subscript letters denote statistically significant differences (*p* = 0.05).

In Figure 5, the biochemical composition, in terms of total protein, total lipids, and total carbohydrates of *Nannochloris* sp. JB17, cultivated with different NaHCO_3_ concentrations in fresh as well as in sea water is depicted. Total protein content fluctuated at close levels among the different treatments (32.4–38.5%), however, the highest protein content was observed in N_60_ cultures. Total lipids showed no significant variation among the different NaHCO_3_ concentrations in both fresh and sea water (12.6–13.3%), while carbohydrate content also ranged at very close levels among the cultures (26–30.9%).

#### 3.2.1. Amino Acids and Protein Digestibility

In Table 1, the profile of the amino acids of *Nannochloris* sp. cultivated with different NaHCO_3_ concentrations in fresh as well as in sea water is shown. In general, the results suggest that there is a consistency of the profile among the different cultures with the exception of only a few amino acids (AAs), i.e., lysine and proline, which their content increased when *Nannochloris* sp. JB17 was cultivated in sea water. Proline and lysine are referred to as important osmoregulatory solutes which significantly reduce the accumulation of Na^+^ and Cl^-^. Moreover, another significant strategy for coping with high salinity involves the efficient uptake and export of ions across cell membranes by the regulation of membrane transport proteins [42]. The total essential AAs ranged between 36 and 40% of the total AAs, while the essential AAs index (EAAI) ranged between 0.85 and 1.02. The cultivation of *Nannochloris* sp. in sea-water-based growing media resulted in a higher content of essential AAs and higher EAAI (0.94–1.02). For human or animal consumption, EAA indices higher than 0.9 are considered of high nutritional value [43].

**Figure 6:**
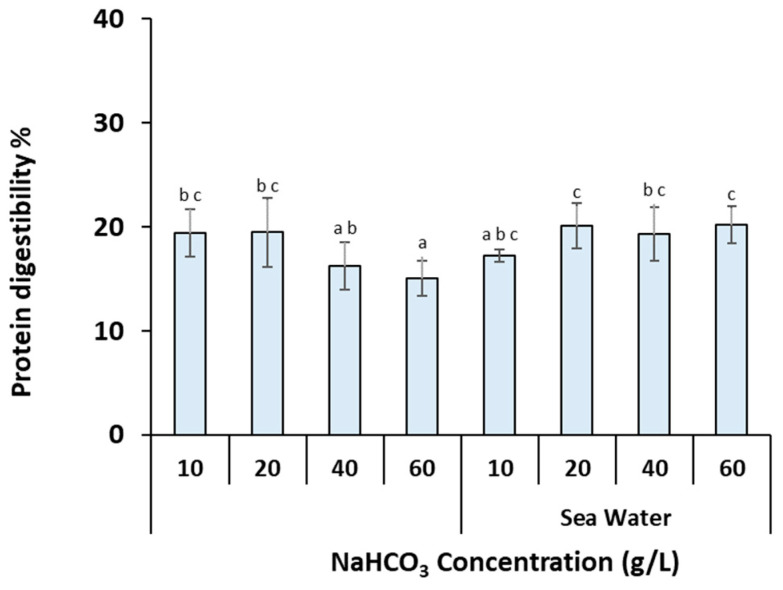
Protein digestibility of *Nannochloris* sp. JB17 according to the different NaHCO_3_ concentrations in fresh as well as in sea water (*n* = 3, ±SD). Different subscript letters denote statistically significant differences (*p* = 0.05).

However, one of the primary parameters which influence the quality of protein is the food matrix digestibility. A greater percentage of protein digestibility indicates also that the proteins are easily accessible to the digestive enzymes and thus the food exhibits elevated nutritional quality [44]. The results illustrated in Figure 6 show that the in vitro protein digestibility of *Nannochloris* sp. JB17 ranged from 15.03 to 20.19% for the different NaHCO_3_ concentrations in fresh as well as in sea water. Compared to other microalgal species, the results show that the in vitro protein digestibility of *Nannochloris* sp. JB17 is significantly lower. In the study of Wild, et al. [45] where 16 microalgal species were assessed, the lowest in vitro protein digestibility found was for Nannochloropsis (54%) and the highest for Chlorella (79%). This low in vitro protein digestibility of *Nannochloris* sp. JB17 is most probable due to the rigid cell wall, which is resistant to the enzymatic cell lysis that reduces protein accessibility [46]. To cope with this, a pre-treatment of biomass to disrupt the cell wall could be a possible way to increase protein accessibility and digestibility [46].

#### 3.2.2. Lipids and Fatty Acid Methyl Esters (FAMEs)

**Figure 7:**
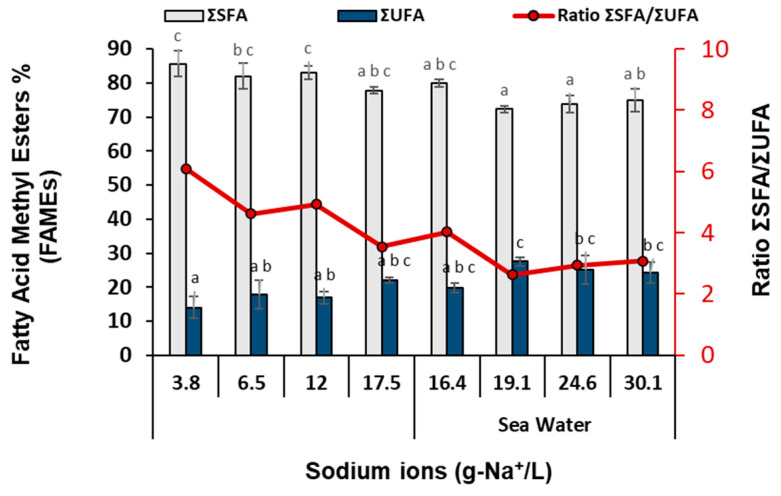
Total saturated (ΣSFA) and unsaturated (ΣUFA) fatty acids and their ratios in *Nannochloris* sp. JB17 according to the different sodium ions concentrations (g-Na^+^/L in fresh as well as in sea water). The data represent the mean of *n* = 3, while error bars represent the standard deviation. Different subscript letters denote statistically significant differences (*p* = 0.05).

Total lipids did not show any significant alteration among the different NaHCO_3_ concentrations in both fresh and sea water (12.6–13.3%; Figure 5). In Table 2, the fatty acid methyl esters (FAMEs) profile of the isolated *Nannochloris* sp. JB17 cultured in different NaHCO_3_ concentrations in fresh as well as in sea water is presented. There is a significant decrease regarding the saturated fatty acid, stearic acid (C18:0), as NaHCO_3_ concentration increased in fresh water, where also a further decrease is observed with the simultaneous presence of salt (from 31.50 to 21.07%), indicating that the overall Na^+^ concentration is a critical parameter. Nervonic acid (C24:1) is also decreased as NaHCO_3_ concentration is increased, while there is no further decrease with the simultaneous presence of salt. a-linolenic acid (C18:3) is significantly increased with the increase of total Na^+^ concentration (1.43 to 4.27%). Although linoleic acid (C18:2) is increased with the increase of total Na^+^ concentration, the highest value is observed for SW_20_ treatment (8.49 to 19.20%) while also myristoleic acid (C14:1), pentadecanoic acid (C15:0), homo-gamma-linolenic acid (C20:3), and behenic acid (C22:0) are significantly increased with the increase of overall Na^+^ concentration. Regarding the fatty acids—myristic acid (C14:0), palmitic acid (C16:0), palmitoleic acid (C16:1), heptadecenoic acid (C17:1), and arachidic acid (C20:0)—no significant differences are observed between the different treatments, while for EPA, EPA, Cis-3 methyl ester (C20:5), heptadecanoic acid (C17:0), and oleic acid Cis-9 (C18:1), no clear trend is observed. Figure 7 depicts the total saturated (ΣSFA) and unsaturated (ΣUFA) fatty acids and their ratio of ΣSFA/ΣUFA according to the different sodium ions concentrations (g-Na^+^/L in fresh as well as in sea water). As shown, there is a positive correlation between the total unsaturated fatty acids and sodium ions. These findings come in agreement with previous studies, which report that under increased NaHCO_3_ concentration (up to 25 g/L), there is a significant increase in total unsaturated lipids, where a-linolenic acid (C18:3) and linoleic acid (C18:2) were significantly increased, with a decrease in total saturated lipids [12]. By altering membrane fatty acid saturation levels, membrane fluidity is influenced which might represent a potential candidate for mitigating the effects of salt stress. The process of fatty acid desaturation might be associated with the activation of a salt-tolerant Na^+^/H^+^ antiporter that is crucial for the removal of Na^+^ [47]. In a prior investigation, it was highlighted that polyunsaturated fatty acids (PUFA) actively contribute to the maintenance of the photosynthetic function. Long-chain PUFA includes essential fatty acids such as linoleic acid and α-linolenic acid, which are crucial for the growth and development of humans. Consequently, *Nannochloris* sp. JB17 holds promise as a raw material for the production of edible oil or health care products [12,48].

#### 3.2.3. Carbohydrates

**Figure 8:**
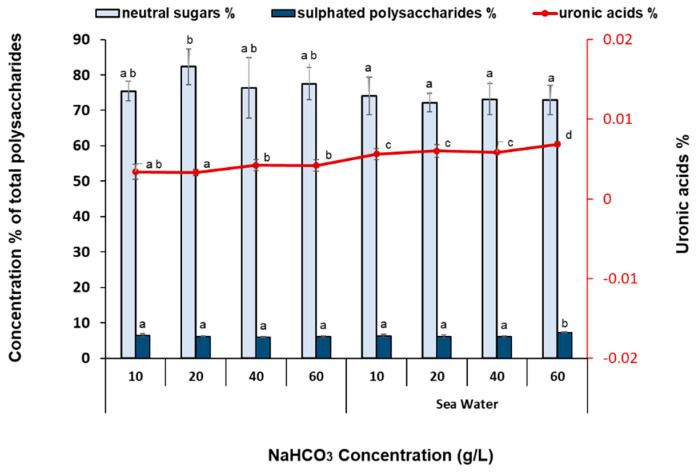
Selected types of sugars (uronic acid and neutral) and sulfated polysaccharides of *Nannochloris* sp. for the cultures treated with different NaHCO_3_ concentrations in fresh as well as in sea water. Percentages are expressed as % of total carbohydrates. The data represent the mean of *n* = 3, while error bars represent the standard deviation. Different subscript letters denote statistically significant differences (*p* = 0.05).

A further characterization of the carbohydrates of *Nannochloris* sp. JB17 cultivated with different NaHCO_3_ concentrations in fresh as well as in sea water is shown in Figure 8. The main fraction of carbohydrates was in the type of neutral sugars ranging from around 72% to 80% (of total carbohydrates), while uronic acids were in negligible amounts. Moreover, *Nannochloris* sp. JB17 showed that it contains around 8–9% sulfated polysaccharides, which is an interesting fraction of carbohydrates since sulfated polysaccharides exert significant biological properties, such as immunity, anti-virus, and anti-oxidation activities [49]. In general, the carbohydrate types in *Nannochloris* sp. JB17 did show consistency and the changes in their cultivation conditions did not affect their content, with the exception of N_20_ where neutral sugars were the highest (statistically significant difference) compared to all other cultures. Nevertheless, as it was shown in Figure 5, *Nannochloris* sp. JB17 contained carbohydrates in the range of 26–31%. This relatively high carbohydrate content along with the integrity of the cell wall in *Nannochloris* sp. JB17 possibly relates to its salt tolerance. Under high salt conditions, numerous carbohydrates are synthesized and accumulated in the chloroplasts and may maintain cellular osmotic balance or be preserved as energy storage compounds to cover cell metabolic energy demands [42].

## 4. Conclusions

In conclusion, *Nannochloris* sp. JB17 displayed a robust growth pattern in both fresh and sea-water-based media with increased bicarbonate concentrations. Only at the higher sodium strength (sea water supplemented with 60 g/L bicarbonates) was the biomass concentration significantly lower, while the best performance was obtained in fresh water media supplemented with 40 g/L and 60 g/L NaHCO_3_. The biochemical composition did not significantly differ between treatments, where total protein content ranged between 32.4 and 38.5%, total lipids between 12.6 and 13.3%, and carbohydrates between 26% and 30.9%. The microalga had a good essential amino acids index of 0.85–1.02 but displayed significantly low in vitro protein digestibility (15–20%). The ratio of the saturated to unsaturated fatty acids ranged between 6 and 3, while the increased sodium strength improved the content of a-linolenic acid (C18:3) and linoleic acid (C18:2). The microalga contained around 8–9% sulfated polysaccharides. The results indicate that *Nannochloris* sp. JB17 is a potential candidate to be used for carbon capture and utilization of biosystems using bicarbonates as the carbon source. However, more research is needed to assess the applicability of the *Nannochloris* sp. JB17 in scaled-up facilities and for further exploration of the produced biomass as food or feed applications.

## Figures and Tables

**Table 1:** Amino Acid (AA) profile. AA percentage of each AA is expressed in relation to the total AAs of the isolated *Nannochloris* sp. JB17 grown on different NaHCO_3_ concentrations in fresh as well as in sea water. The data represent the mean of *n* = 3 ± standard deviation. Different subscript letters denote statistically significant differences (*p* = 0.05).

Amino Acid	N_10_ (%)	N_20_ (%)	N_40_ (%)	N_60_ (%)	SW_10_ (%)	SW_20_ (%)	SW_40_ (%)	SW_60_ (%)
Aspartic acid	6.49 ± 0.66 ^a^	6.82 ± 1.28 ^a^	6.51 ± 0.51 ^a^	6.46 ± 0.38 ^a^	5.90 ± 0.14 ^a^	5.88 ± 0.54 ^a^	5.91 ± 0.79 ^a^	5.61 ± 0.42 ^a^
Glutamic acid	10.90 ± 1.04 ^a^	11.38 ± 1.48 ^a^	11.07 ± 0.86 ^a^	11.03 ± 0.49 ^a^	10.04 ± 0.32 ^a^	10.06 ± 0.97 ^a^	9.98 ± 0.90 ^a^	9.74 ± 0.65 ^a^
Serine	4.53 ± 0.18 ^a^	4.54 ± 0.17 ^a^	4.46 ± 0.15 ^a^	4.44 ± 0.05 ^a^	4.63 ± 0.42 ^a^	4.70 ± 0.69 ^a^	4.19 ± 0.14 ^a^	4.04 ± 0.23 ^a^
Histidine	3.57 ± 0.82 ^a^	3.32 ± 0.88 ^a^	3.37 ± 0.24 ^a^	3.27 ± 0.43 ^a^	3.43 ± 0.42 ^a^	3.53 ± 0.49 ^a^	3.40 ± 0.82 ^a^	3.32 ± 0.35 ^a^
Glycine	12.61 ± 2.32 ^a^	11.74 ± 2.86 ^a^	11.74 ± 0.91 ^a^	11.49 ± 1.29 ^a^	11.32 ± 0.53 ^a^	11.09 ± 2.04 ^a^	10.75 ± 3.00 ^a^	10.34 ± 1.75 ^a^
Threonine	3.91 ± 0.52 ^a^	4.22 ± 0.62 ^a^	4.14 ± 0.38 ^a^	3.95 ± 0.44 ^a^	3.74 ± 0.14 ^a^	3.72 ± 0.40 ^a^	3.58 ± 0.45 ^a^	3.55 ± 0.37 ^a^
Alginine + Alanine	20.08 ± 0.75 ^b^	19.60 ± 1.23 ^ab^	19.65 ± 0.33 ^ab^	19.34 ± 0.50 ^ab^	18.50 ± 0.81 ^ab^	18.08 ± 0.32 ^ab^	17.49 ± 1.84 ^a^	17.57 ± 1.79 ^ab^
Tyrosine	3.09 ± 0.84 ^a^	2.17 ± 1.81 ^a^	3.31 ± 0.24 ^a^	3.29 ± 0.26 ^a^	2.63 ± 1.80 ^a^	3.62 ± 1.39 ^a^	3.45 ± 0.23 ^a^	3.32 ± 0.19 ^a^
Cysteine	0.32 ± 0.02 ^a^	0.29 ± 0.02 ^a^	0.22 ± 0.04 ^a^	0.42 ± 0.08 ^a^	0.23 ± 0.04 ^a^	0.21 ± 0.06 ^a^	0.35 ± 0.08 ^a^	0.30 ± 0.10 ^a^
Valine	5.64 ± 0.24 ^a^	5.72 ± 0.42 ^a^	5.67 ± 0.40 ^a^	5.81 ± 0.15 ^a^	5.41 ± 0.10 ^a^	5.35 ± 0.27 ^a^	5.24 ± 0.25 ^a^	5.22 ± 0.33 ^a^
Methionine	2.67 ± 0.80 ^a^	4.20 ± 1.53 ^a^	4.22 ± 2.46 ^a^	2.88 ± 1.15 ^a^	1.84 ± 1.08 ^a^	3.39 ± 0.15 ^a^	5.59 ± 2.25 ^a^	5.46 ± 2.46 ^a^
Phenylalanine	6.61 ± 0.25 ^b^	6.34 ± 0.38 ^ab^	6.29 ± 0.25 ^ab^	6.45 ± 0.29 ^ab^	6.29 ± 0.12 ^ab^	5.96 ± 0.50 ^ab^	5.77 ± 0.21 ^ab^	5.58 ± 0.25 ^a^
Isoleucine	4.62 ± 0.10 ^a^	4.63 ± 0.11 ^a^	4.51± 0.12 ^a^	4.55 ± 0.29 ^a^	4.68 ± 0.18 ^a^	4.62 ± 0.10 ^a^	4.59 ± 0.06 ^a^	4.47 ± 0.25 ^a^
Leucine	10.72 ± 0.10 ^a^	10.47 ± 0.32 ^a^	10.32 ± 0.41 ^a^	10.83 ± 0.93 ^a^	11.26 ± 0.53 ^a^	10.77 ± 0.51 ^a^	10.48 ± 0.38 ^a^	10.32 ± 0.44 ^a^
Lysine	1.77 ± 0.53 ^a^	1.38 ± 0.14 ^a^	1.42 ± 0.36 ^a^	2.59 ± 2.01 ^ab^	5.50 ± 1.47 ^b^	4.28 ± 0.05 ^ab^	3.89 ± 0.77 ^ab^	4.13 ± 0.13 ^ab^
Proline	2.47 ± 1.02 ^a^	3.18 ± 1.90 ^a^	3.11 ± 0.46 ^a^	4.67 ± 1.53 ^a^	4.57 ± 2.39 ^a^	4.72 ± 1.58 ^a^	5.34 ± 3.28 ^a^	4.84 ± 1.86 ^a^
ΣΕAAs *	35.93 ± 1.33 ^a^	36.90 ± 2.21 ^a^	36.53± 1.32 ^a^	37.13 ± 2.15 ^a^	38.73 ± 3.04 ^a^	38.13 ± 1.85 ^a^	39.13 ± 1.22 ^a^	39.53 ± 2.41 ^a^
EAAs index	0.85	0.86	0.86	0.89	0.94	0.97	1.01	1.02

* ΣΕAAs = sum of essential amino acids.

**Table 2:** Fatty Acid Methyl Esters (FAMEs). Fatty Acid Methyl Esters of the isolated *Nannochloris* sp. grown on different NaHCO_3_ concentrations in fresh as well as in sea water. The data represent the mean of *n* = 3, while error bars represent the standard deviation. Different subscript letters denote statistically significant differences (*p* = 0.05).

Fatty Acid Methyl Esters (FAMEs)	Fresh Water				Sea water			
	10 g/L NaHCO_3_	20 g/L NaHCO_3_	40 g/L NaHCO_3_	60 g/L NaHCO_3_	10 g/L NaHCO_3_	20 g/L NaHCO_3_	40 g/L NaHCO_3_	60 g/L NaHCO_3_
C14:0 Myristic acid methyl ester	1.43 ± 0.34 ^a^	1.22 ± 0.39 ^a^	1.33 ± 0.18 ^a^	1.30 ± 0.07 ^a^	1.32 ± 0.15 ^a^	1.13 ± 0.14 ^a^	1.37 ± 0.36 ^a^	1.19 ± 0.11 ^a^
C14:1 Myristoleic acid, Cis-9 methyl ester	0.52 ± 0.09 ^a^	0.85 ± 0.27 ^b^	0.52 ± 0.03 ^a^	0.61 ± 0.03 ^a b^	0.70 ± 0.08 ^a b^	1.44 ± 0.16 ^b c^	2.04 ± 0.24 ^c^	1.96 ± 0.28 ^c^
C15:0 Pentadecanoic acid methyl ester	0.07 ± 0.02 ^a^	0.05 ± 0.02 ^a^	0.27 ± 0.04 ^a^	0.37 ± 0.01 ^b^	0.07 ± 0.01 ^a^	1.17 ± 0.11 ^b c^	1.56 ± 0.16 ^c^	1.43 ± 0.29 ^b c^
C16:0 Palmitic acid methyl ester	51.61 ± 2.75 ^a^	49.54 ± 3.72 ^a^	50.59 ± 1.47 ^a^	48.86 ± 1.21 ^a^	49.24 ± 1.63 ^a^	47.94 ± 2.02 ^a^	46.38 ± 0.89 ^a^	47.72 ± 1.53 ^a^
C16:1 Palmitoleic acid methyl ester	0.45 ± 0.06 ^a^	0.51 ± 0.15 ^a^	0.36 ± 0.06 ^a^	0.42 ± 0.02 ^a^	0.41 ± 0.03 ^a^	0.43 ± 0.05 ^a^	0.53 ± 0.02 ^a^	0.55 ± 0.09 ^a^
C17:0 Heptadecanoic acid methyl ester	0.51 ± 0.04 ^a b^	0.57 ± 0.15 ^a b^	0.33 ± 0.15 ^a^	0.45 ± 0.06 ^a b^	0.42 ± 0.09 ^a^	0.60 ± 0.04 ^a b^	0.72 ± 0.07 ^b^	0.48 ± 0.15 ^a b^
C17:1 Heptadecenoic acid methyl ester	0.15 ± 0.07 ^a^	0.24 ± 0.00 ^a^	0.15 ± 0.04 ^a^	0.14 ± 0.02 ^a^	0.19 ± 0.03 ^a^	0.20 ± 0.09 ^a^	0.23 ± 0.10 ^a^	0.14 ± 0.07 ^a^
C18:0 Stearic acid methyl ester	31.50 ± 5.68 ^c^	30.12 ± 2.97 ^b c^	30.01 ± 1.16 ^b c^	26.36 ± 0.90 ^a b c^	28.36 ± 3.00 ^a b c^	21.07 ± 0.47 ^a^	22.98 ± 2.09 ^a b^	23.42 ± 2.05 ^a b c^
C18:1 Oleic acid, Cis-9 methyl ester	2.26 ± 0.38 ^a b^	1.90 ± 0.51 ^a^	1.89 ± 0.28 ^a^	2.54 ± 0.16 ^a b^	2.34 ± 0.46 ^a b^	3.62 ± 0.92 ^b^	2.36 ± 0.53 ^a b^	2.49 ± 0.26 ^a b^
C18:2 Linoleic acid, Cis-9 methyl ester	8.49 ± 2.43 ^a^	11.51 ± 3.26 ^a b c^	11.16 ± 1.47 ^a b^	14.53 ± 0.59 ^b c^	12.70 ± 0.51 ^a b c^	19.20 ± 2.80 ^c^	14.67 ± 1.89 ^b c^	13.94 ± 1.56 ^a b c^
C20:0 Arachidic acid methyl ester	0.31 ± 0.04 ^a^	0.31 ± 0.07 ^a^	0.32 ± 0.06 ^a^	0.32 ± 0.03 ^a^	0.34 ± 0.04 ^a^	0.30 ± 0.09 ^a^	0.23 ± 0.09 ^a^	0.17 ± 0.04 ^a^
C18:3 a-Linolenic acid, Cis-3 methyl ester	1.43 ± 0.44 ^a^	2.11 ± 0.72 ^a b^	2.23 ± 0.41 ^a b^	2.97 ± 0.18 ^a b c^	2.71 ± 0.37 ^b c d^	3.83 ± 0.36 ^c d^	3.48 ± 0.54 ^b c d^	4.27 ± 0.92 ^d^
C20:3 Homo-gamma-Linolenic acid, Cis-6 methyl ester	-	-	0.15 ± 0.06 ^a^	0.20 ± 0.01 ^a^	0.21 ± 0.04 ^a^	0.50 ± 0.13 ^b^	0.52 ± 0.16 ^b^	0.49 ± 0.10 ^b^
C22:0 Behenic acid methyl ester	0.23 ± 0.06 ^a b c^	0.25 ± 0.09 ^a b^	0.30 ± 0.07 ^a b c^	0.18 ± 0.12 ^a^	0.31 ± 0.01 ^a b c^	0.59 ± 0.21 ^b c^	0.63 ± 0.14 ^c^	0.51 ± 0.08 ^a b c^
C20:5 EPA, Cis-3 methyl ester	0.39 ± 0.09 ^c d^	0.48 ± 0.13 ^c d e^	0.17 ± 0.07 ^a b c^	0.10 ± 0.02 ^a b^	0.07 ± 0.01 ^a^	0.98 ± 0.35 ^d e^	1.14 ± 0.47 ^e^	0.32 ± 0.06 ^b c^
C24:1 Nervonic acid, Cis-9 methyl ester	0.63 ± 0.10 ^a b^	0.51 ± 0.01 ^a b^	0.45 ± 0.10 ^a^	0.51 ± 0.07 ^a b^	0.82 ± 0.03 ^b^	0.55 ± 0.02 ^a b^	0.48 ± 0.06 ^a^	0.52 ± 0.20 ^a b^

## Data Availability

The original contributions presented in the study are included in the article/Supplementary Material, further inquiries can be directed to the corresponding author.

## Supplementary Materials

The following supporting information can be downloaded at: https://www.mdpi.com/article/10.3390/bioengineering11121301/s1, Section S1: Adaptation protocol, Section S2: detailed statistical analyses of the results.

## References

[1] Mobin S.M.A., Chowdhury H., Alam F. (2019). Commercially important bioproducts from microalgae and their current applications—A review. Energy Procedia.

[2] Daneshvar E., Wicker R.J., Show P.-L., Bhatnagar A. (2022). Biologically-mediated carbon capture and utilization by microalgae towards sustainable CO_2_ biofixation and biomass valorization—A review. Chem. Eng. J..

[3] Cui X., Yang J., Cui M., Zhang W., Zhao J. (2021). Comparative experiments of two novel tubular photobioreactors with an inner aerated tube for microalgal cultivation: Enhanced mass transfer and improved biomass yield. Algal Res..

[4] Nguyen L.N., Vu M.T., Vu H.P., Johir M.A.H., Labeeuw L., Ralph P.J., Mahlia T., Pandey A., Sirohi R., Nghiem L.D. (2023). Microalgae-based carbon capture and utilization: A critical review on current system developments and biomass utilization. Crit. Rev. Environ. Sci. Technol..

[5] Zheng Q., Xu X., Martin G.J.O., Kentish S.E. (2018). Critical review of strategies for CO_2_ delivery to large-scale microalgae cultures. Chin. J. Chem. Eng..

[6] Zhu C., Chen S., Ji Y., Schwaneberg U., Chi Z. (2022). Progress toward a bicarbonate-based microalgae production system. Trends Biotechnol..

[7] Kim G.-Y., Roh K., Han J.-I. (2019). The use of bicarbonate for microalgae cultivation and its carbon footprint analysis. Green Chem..

[8] Chi Z., Elloy F., Xie Y., Hu Y., Chen S. (2014). Selection of Microalgae and Cyanobacteria Strains for Bicarbonate-Based Integrated Carbon Capture and Algae Production System. Appl. Biochem. Biotechnol..

[9] Zhang R.-L., Wang J.-H., Cheng L.-Y., Tang Y.-J., Chi Z.-Y. (2019). Selection of microalgae strains for bicarbonate-based integrated carbon capture and algal production system to produce lipid. Int. J. Green Energy.

[10] Singh J., Maharana C., Dhar D.W. (2022). Alkalihalophilic alga *Picocystis salinarum* SLJS6 from Sambhar Salt Lake: Potential for bicarbonate-based biomass production and carbon capture. Bioresour. Technol. Rep..

[11] Piiparinen J., Barth D., Eriksen N.T., Teir S., Spilling K., Wiebe M.G. (2018). Microalgal CO_2_ capture at extreme pH values. Algal Res..

[12] Wang M., Liu H., Qiao K., Ye X., Takano T., Liu S., Bu Y. (2021). Exogenous NaHCO_3_ enhances growth and lipid accumulation of the highly NaHCO_3_-tolerant *Nannochloris* sp. JB17. J. Appl. Phycol..

[13] Zhu C., Zhang R., Cheng L., Chi Z. (2018). A recycling culture of Neochloris oleoabundans in a bicarbonate-based integrated carbon capture and algae production system with harvesting by auto-flocculation. Biotechnol. Biofuels.

[14] Wang M., Ye X., Wang Y., Su D., Liu S., Bu Y. (2021). Transcriptome dynamics and hub genes of green alga *Nannochloris* sp. JB17 under NaHCO_3_ stress. Algal Res..

[15] Qiao K., Takano T., Liu S. (2015). Discovery of two novel highly tolerant NaHCO_3_ Trebouxiophytes: Identification and characterization of microalgae from extreme saline–alkali soil. Algal Res..

[16] Zhu Z., Jiang J., Fa Y. (2020). Overcoming the biological contamination in microalgae and cyanobacteria mass cultivations for photosynthetic biofuel production. Molecules.

[17] Lowry O.H., Rosebrough N.J., Farr A.L., Randall R.J. (1951). Protein measurement with the Folin phenol reagent. J. Biol. Chem..

[18] Kochert G., Hellebust J.A., Craige J.S. (1978). Carbohydrate determination by phenol-sulfuric acid method. Handbook of Phycological Methods. Physiological and Biochemical Methods.

[19] Mishra S.K., Suh W.I., Farooq W., Moon M., Shrivastav A., Park M.S., Yang J.-W. (2014). Rapid quantification of microalgal lipids in aqueous medium by a simple colorimetric method. Bioresour. Technol..

[20] Markou G., Eliopoulos C., Argyri A., Arapoglou D. (2021). Production of Arthrospira (Spirulina) platensis Enriched in β-Glucans through Phosphorus Limitation. Appl. Sci..

[21] Lichtenthaler H.K. (1987). [34] Chlorophylls and carotenoids: Pigments of photosynthetic biomembranes. Methods Enzymology.

[22] Goiris K., Muylaert K., Fraeye I., Foubert I., De Brabanter J., De Cooman L. (2012). Antioxidant potential of microalgae in relation to their phenolic and carotenoid content. J. Appl. Phycol..

[23] Hajimahmoodi M., Faramarzi M.A., Mohammadi N., Soltani N., Oveisi M.R., Nafissi-Varcheh N. (2010). Evaluation of antioxidant properties and total phenolic contents of some strains of microalgae. J. Appl. Phycol..

[24] Li H.-B., Cheng K.-W., Wong C.-C., Fan K.-W., Chen F., Jiang Y. (2007). Evaluation of antioxidant capacity and total phenolic content of different fractions of selected microalgae. Food Chem..

[25] Almeida C.C., Monteiro M.L.G., da Costa-Lima B.R.C., Alvares T.S., Conte-Junior C.A. (2015). In vitro digestibility of commercial whey protein supplements. LWT-Food Sci. Technol..

[26] Lie S. (1973). The EBC-ninhydrin method for determination of free alpha amino nitrogen. J. Inst. Brew..

[27] Oser B.L., Albanese A. (1959). An integrated essential amino acid index for predicting the biological value of proteins. Protein and Amino Acid Nutrition.

[28] Monsigny M., Petit C., Roche A.-C. (1988). Colorimetric determination of neutral sugars by a resorcinol sulfuric acid micromethod. Anal. Biochem..

[29] Blumenkrantz N., Asboe-Hansen G. (1973). New method for quantitative determination of uronic acids. Anal. Biochem..

[30] Dodgson K., Price R. (1962). A note on the determination of the ester sulphate content of sulphated polysaccharides. Biochem. J..

[31] Liu C., Liu J., Hu S., Wang X., Wang X., Guan Q. (2019). Isolation and identification of a halophilic and alkaliphilic microalgal strain. PeerJ.

[32] PSI Operation Manual. https://handheld.psi.cz/products/aquapen-c-and-aquapen-p/.

[33] McMurdie P.J., Holmes S. (2013). phyloseq: An R package for reproducible interactive analysis and graphics of microbiome census data. PLoS ONE.

[34] Murchie E.H., Lawson T. (2013). Chlorophyll fluorescence analysis: A guide to good practice and understanding some new applications. J. Exp. Bot..

[35] Masojídek J., Vonshak A., Torzillo G. (2010). Chlorophyll fluorescence applications in microalgal mass cultures. Chlorophyll a Fluorescence in Aquatic Sciences: Methods and Applications.

[36] Kromdijk J., Walter J., Sharwood R. (2023). Relaxing non-photochemical quenching (NPQ) to improve photosynthesis in crops. Understanding and Improving Crop Photosynthesis.

[37] Ruban A.V. (2017). Quantifying the efficiency of photoprotection. Philos. Trans. R. Soc. B Biol. Sci..

[38] Borowitzka M.A. (2018). The ‘stress’ concept in microalgal biology—Homeostasis, acclimation and adaptation. J. Appl. Phycol..

[39] Sosnowski J., Truba M. (2021). Photosynthetic activity and chlorophyll pigment concentration in *Medicago* x varia T. Martyn leaves treated with the Tytanit growth regulator. Saudi J. Biol. Sci..

[40] Caferri R., Guardini Z., Bassi R., Dall’Osto L., Wurtzel E.T. (2022). Chapter Two—Assessing photoprotective functions of carotenoids in photosynthetic systems of plants and green algae. Methods Enzymology.

[41] Xiao F.-G., Shen L., Ji H.-F. (2011). On photoprotective mechanisms of carotenoids in light harvesting complex. Biochem. Biophys. Res. Commun..

[42] Shetty P., Gitau M.M., Maróti G. (2019). Salinity stress responses and adaptation mechanisms in eukaryotic green microalgae. Cells.

[43] Tibbetts S.M., Milley J.E., Lall S.P. (2015). Chemical composition and nutritional properties of freshwater and marine microalgal biomass cultured in photobioreactors. J. Appl. Phycol..

[44] Fleurence J. (1999). Seaweed proteins: Biochemical, nutritional aspects and potential uses. Trends Food Sci. Technol..

[45] Wild K.J., Steingass H., Rodehutscord M. (2018). Variability in nutrient composition and in vitro crude protein digestibility of 16 microalgae products. J. Anim. Physiol. Anim. Nutr..

[46] Van De Walle S., Broucke K., Baune M.-C., Terjung N., Van Royen G., Boukid F. (2024). Microalgae protein digestibility: How to crack open the black box?. Crit. Rev. Food Sci. Nutr..

[47] Henley W.J., Major K.M., Hironaka J.L. (2002). Response to salinity and heat stress in two halotolerant chlorophyte algae. J. Phycol..

[48] Wang K.S., Chai T.-J. (1994). Reduction in omega-3 fatty acids by UV-B irradiation in microalgae. J. Appl. Phycol..

[49] Huang L., Shen M., Morris G.A., Xie J. (2019). Sulfated polysaccharides: Immunomodulation and signaling mechanisms. Trends Food Sci. Technol..

