# Supplementary material for: Nannochloris sp. JB17 as a Potential Microalga for Carbon Capture and Utilization Bio-Systems: Growth and Biochemical Composition Under High Bicarbonate Concentrations in Fresh and Sea Water
Source: Bioengineering (Basel). 2024 Dec 23;11(12):1301. doi: 10.3390/bioengineering11121301 (PMC11727422; doi:10.3390/bioengineering11121301)
Supplement: Supplementary file 1 [file bioengineering-11-01301-s001.zip › bioengineering-3297749-supplementary.pdf]

Supplementary material

Nannochloris sp. JB17 as a potential microalga for carbon capture and utilization bio-systems: Growth and biochemical composition under high bicarbonate concentrations in fresh and sea-water

Giorgos Markou[1*], Eleni Kougia[1] and Dimitris Arapoglou[1]

[1] Institute of Technology of Agricultural Products, ELGO - Dimitra, Leof. Sofokli Venizelou 1, Lykovrysi, 14123 Athens, Greece
* Correspondence: markougior@elgo.gr

In this supplementary material, the adaptation procedure followed prior conducting the experiments is illustrated (Section S1) and the statistical analysis of the presented results of the manuscript are given in more detail (Section S2).

Section S1

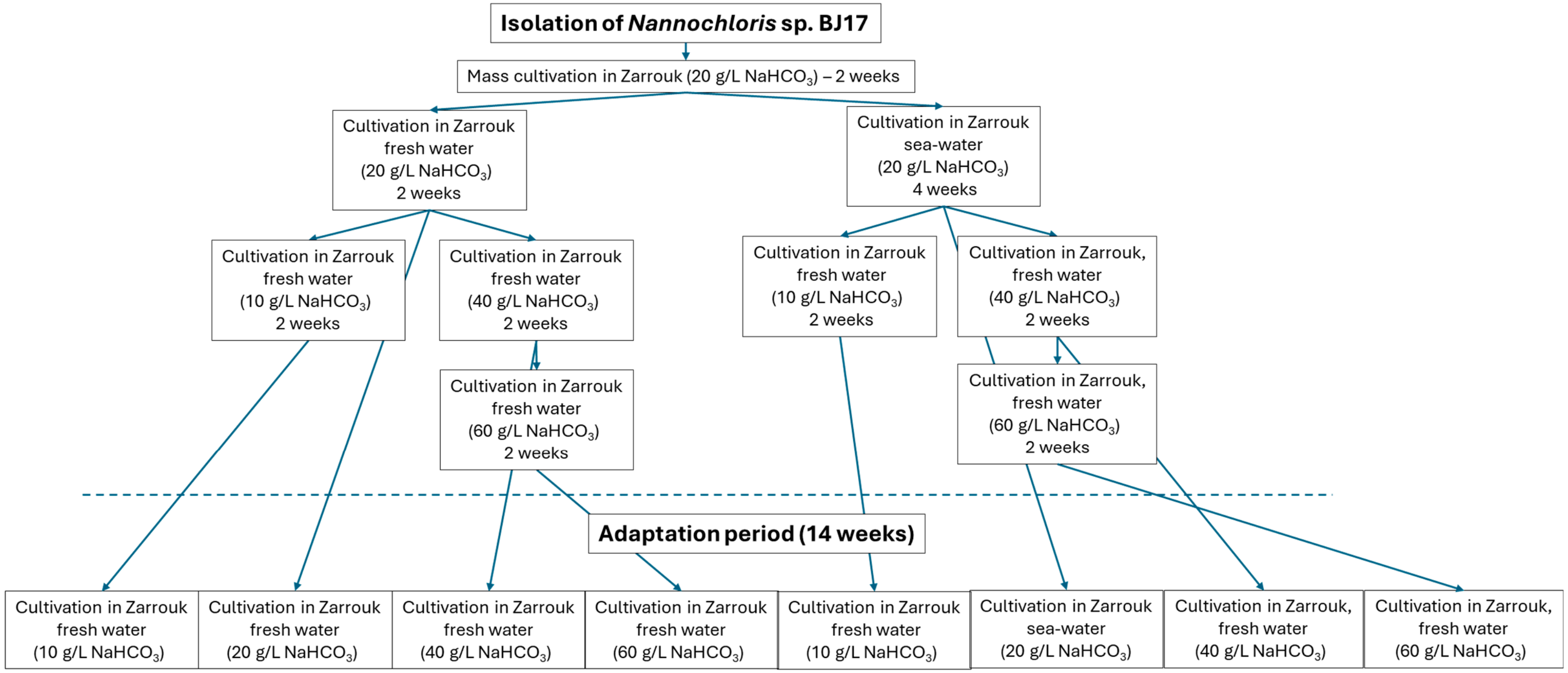

Isolation of *Nannochloris* sp. BJ17
Mass cultivation in Zarrouk (20 g/L $NaHCO_3$) – 2 weeks
Cultivation in Zarrouk fresh water (20 g/L $NaHCO_3$) 2 weeks
Cultivation in Zarrouk sea-water (20 g/L $NaHCO_3$) 4 weeks
Cultivation in Zarrouk fresh water (10 g/L $NaHCO_3$) 2 weeks
Cultivation in Zarrouk fresh water (40 g/L $NaHCO_3$) 2 weeks
Cultivation in Zarrouk fresh water (10 g/L $NaHCO_3$) 2 weeks
Cultivation in Zarrouk, fresh water (40 g/L $NaHCO_3$) 2 weeks
Cultivation in Zarrouk fresh water (60 g/L $NaHCO_3$) 2 weeks
Cultivation in Zarrouk, fresh water (60 g/L $NaHCO_3$) 2 weeks
Adaptation period (14 weeks)
Cultivation in Zarrouk fresh water (10 g/L $NaHCO_3$)
Cultivation in Zarrouk fresh water (20 g/L $NaHCO_3$)
Cultivation in Zarrouk fresh water (40 g/L $NaHCO_3$)
Cultivation in Zarrouk fresh water (60 g/L $NaHCO_3$)
Cultivation in Zarrouk fresh water (10 g/L $NaHCO_3$)
Cultivation in Zarrouk sea-water (20 g/L $NaHCO_3$)
Cultivation in Zarrouk, fresh water (40 g/L $NaHCO_3$)
Cultivation in Zarrouk, fresh water (60 g/L $NaHCO_3$)

Section S2

Dry weight

2 way anova

Tests of Between-Subjects Effects

Dependent Variable:

Source	Type III Sum of Squares	df	Mean Square	F	Sig.	Partial Eta Squared
Corrected Model	,674[a]	4	,168	7,725	,000	,418
Intercept	56,615	1	56,615	2595,915	,000	,984
nahco3	,206	3	,069	3,146	,035	,180
salt	,468	1	,468	21,462	,000	,333
Error	,938	43	,022			
Total	58,227	48				
Corrected Total	1,612	47				

a. R Squared = ,418 (Adjusted R Squared = ,364)

Pairwise Comparisons

Dependent Variable:

(I) nahco3		Mean Difference (I-J)	Std. Error	Sig.[b]	95% Confidence Interval for Difference[b]	
					Lower Bound	Upper Bound
10,00	20,00	-,008	,060	,891	-,130	,113
	40,00	-,094	,060	,127	-,215	,028
	60,00	,091	,060	,137	-,030	,213
20,00	10,00	,008	,060	,891	-,113	,130
	40,00	-,085	,060	,164	-,207	,036
	60,00	,100	,060	,106	-,022	,221

40,00	10,00	,094	,060	,127	-,028	,215
	20,00	,085	,060	,164	-,036	,207
	60,00	,185*	,060	,004	,063	,307
60,00	10,00	-,091	,060	,137	-,213	,030
	20,00	-,100	,060	,106	-,221	,022
	40,00	-,185*	,060	,004	-,307	-,063

Based on estimated marginal means

*. The mean difference is significant at the ,05 level.

b. Adjustment for multiple comparisons: Least Significant Difference (equivalent to no adjustments).

Pairwise Comparisons

Dependent Variable:

(I) salt		Mean Difference (I-J)	Std. Error	Sig.[b]	95% Confidence Interval for Difference[b]	
					Lower Bound	Upper Bound
,00	30,00	,197*	,043	,000	,112	,283
30,00	,00	-,197*	,043	,000	-,283	-,112

Based on estimated marginal means

*. The mean difference is significant at the ,05 level.

b. Adjustment for multiple comparisons: Least Significant Difference (equivalent to no adjustments).

Multiple Comparisons

Dependent Variable:

Tukey HSD

(I) nahco3		Std. Error	Sig.	95% Confidence Interval

		Mean Difference (I-J)			Lower Bound	Upper Bound
10,00	20,00	-,0083	,06029	,999	-,1695	,1528
	40,00	-,0938	,06029	,415	-,2549	,0674
	60,00	,0913	,06029	,438	-,0699	,2524
20,00	10,00	,0083	,06029	,999	-,1528	,1695
	40,00	-,0854	,06029	,496	-,2465	,0757
	60,00	,0996	,06029	,361	-,0615	,2607
40,00	10,00	,0938	,06029	,415	-,0674	,2549
	20,00	,0854	,06029	,496	-,0757	,2465
	60,00	,1850*	,06029	,019	,0239	,3461
60,00	10,00	-,0913	,06029	,438	-,2524	,0699
	20,00	-,0996	,06029	,361	-,2607	,0615
	40,00	-,1850*	,06029	,019	-,3461	-,0239

Based on observed means.
The error term is Mean Square(Error) = ,022.
*. The mean difference is significant at the ,05 level.

Homogeneous Subsets

data

Tukey HSD[a,b]

nahco3	N	Subset 1	Subset 2
60,00	12	,9921	
10,00	12	1,0833	1,0833
20,00	12	1,0917	1,0917
40,00	12		1,1771

Sig.		,361	,415

Means for groups in homogeneous subsets are displayed.
Based on observed means.
The error term is Mean Square(Error) = ,022.

a. Uses Harmonic Mean Sample Size = 12,000.

b. Alpha = ,05.

Proteins

Tests of Between-Subjects Effects

Dependent Variable:

Source	Type III Sum of Squares	df	Mean Square	F	Sig.	Partial Eta Squared
Corrected Model	149,126[a]	7	21,304	1,843	,094	,168
Intercept	84263,546	1	84263,546	7290,186	,000	,991
NaHCO3	73,849	3	24,616	2,130	,105	,091
salinity	,926	1	,926	,080	,778	,001
NaHCO3 * salinity	74,350	3	24,783	2,144	,103	,091
Error	739,743	64	11,558			
Total	85152,415	72				
Corrected Total	888,869	71				

a. R Squared = ,168 (Adjusted R Squared = ,077)

Carbohydrates

Tests of Between-Subjects Effects

Dependent Variable:

Source	Type III Sum of Squares	df	Mean Square	F	Sig.	Partial Eta Squared
Corrected Model	141,575[a]	7	20,225	5,030	,000	,362
Intercept	57727,423	1	57727,423	14355,922	,000	,996
NaHCO3	84,377	3	28,126	6,994	,000	,253
salinity	21,284	1	21,284	5,293	,025	,079
NaHCO3 * salinity	34,342	3	11,447	2,847	,045	,121
Error	249,312	62	4,021			
Total	58664,609	70				
Corrected Total	390,887	69				

a. R Squared = ,362 (Adjusted R Squared = ,290)

Pairwise Comparisons

Dependent Variable:

(I) salinity		Mean Difference (I-J)	Std. Error	Sig.[b]	95% Confidence Interval for Difference[b]	
					Lower Bound	Upper Bound
,00	30,00	1,107*	,481	,025	,145	2,068

30,00	,00	-1,107*	,481	,025	-2,068	-,145

Based on estimated marginal means

*. The mean difference is significant at the ,05 level.

b. Adjustment for multiple comparisons: Least Significant Difference (equivalent to no adjustments).

data

Tukey HSD[a,b,c]

NaHCO3	N	Subset		
		1	2	3
4,00	18	27,3959		
1,00	16	28,2198	28,2198	
2,00	18		29,6508	29,6508
3,00	18			30,0742
Sig.		,620	,162	,924

Means for groups in homogeneous subsets are displayed.
Based on observed means.
The error term is Mean Square(Error) = 4,021.
a. Uses Harmonic Mean Sample Size = 17,455.

b. The group sizes are unequal. The harmonic mean of the group sizes is used. Type I error levels are not guaranteed.
c. Alpha = ,05.

Lipids

Tests of Between-Subjects Effects

Dependent Variable:

Source	Type III Sum of Squares	df	Mean Square	F	Sig.	Partial Eta Squared
Corrected Model	3,405[a]	7	,486	1,158	,339	,112
Intercept	11989,067	1	11989,067	28540,185	,000	,998
NaHCO3	,038	3	,013	,030	,993	,001
salinity	2,074	1	2,074	4,938	,030	,072
NaHCO3 * salinity	1,293	3	,431	1,026	,387	,046
Error	26,885	64	,420			
Total	12019,358	72				
Corrected Total	30,290	71				

a. R Squared = ,112 (Adjusted R Squared = ,015)

Pairwise Comparisons

Dependent Variable:

(I) salinity		Mean Difference (I-J)	Std. Error	Sig.[b]	95% Confidence Interval for Difference[b]	
					Lower Bound	Upper Bound
,00	30,00	-,339*	,153	,030	-,645	-,034
30,00	,00	,339*	,153	,030	,034	,645

Based on estimated marginal means

*. The mean difference is significant at the ,05 level.

b. Adjustment for multiple comparisons: Least Significant Difference (equivalent to no adjustments).

PHENOLICS -ABTS ANTIOX CAPACITY

Tests of Between-Subjects Effects

Dependent Variable:

Source	Type III Sum of Squares	df	Mean Square	F	Sig.	Partial Eta Squared
Corrected Model	14,467[a]	7	2,067	84,597	,000	,904
Intercept	279,986	1	279,986	11460,755	,000	,995
NaHCO3	1,705	3	,568	23,270	,000	,526
salinity	9,911	1	9,911	405,710	,000	,866
NaHCO3 * salinity	2,680	3	,893	36,563	,000	,635
Error	1,539	63	,024			
Total	297,307	71				
Corrected Total	16,006	70				

a. R Squared = ,904 (Adjusted R Squared = ,893)

Pairwise Comparisons

Dependent Variable:

(I) salinity		Mean Difference (I-J)	Std. Error	Sig.[b]	95% Confidence Interval for Difference[b] Lower Bound	Upper Bound
,00	30,00	-,748*	,037	,000	-,822	-,674
30,00	,00	,748*	,037	,000	,674	,822

Based on estimated marginal means

*. The mean difference is significant at the ,05 level.

b. Adjustment for multiple comparisons: Least Significant Difference (equivalent to no adjustments).

data

Tukey HSD[a,b,c]

NaHCO3	N	Subset 1	2
1,00	18	1,8712	
3,00	17	1,9124	
2,00	18	1,9213	
4,00	18		2,2527
Sig.		,775	1,000

Means for groups in homogeneous subsets are displayed.
Based on observed means.
The error term is Mean Square(Error) = ,024.
a. Uses Harmonic Mean Sample Size = 17,739.

b. The group sizes are unequal. The harmonic mean of the group sizes is used. Type I error levels are not guaranteed.
c. Alpha = ,05.

Chlorophyll a

Tests of Between-Subjects Effects

Dependent Variable:

Source	Type III Sum of Squares	df	Mean Square	F	Sig.	Partial Eta Squared
Corrected Model	1,672[a]	7	,239	105,500	,000	,949
Intercept	15,292	1	15,292	6755,997	,000	,994
Nahco3	,244	3	,081	35,878	,000	,729
salinity	1,097	1	1,097	484,820	,000	,924
Nahco3 * salinity	,331	3	,110	48,682	,000	,785
Error	,091	40	,002			
Total	17,054	48				
Corrected Total	1,762	47				

a. R Squared = ,949 (Adjusted R Squared = ,940)

Pairwise Comparisons

Dependent Variable:

(I) salinity		Mean Difference (I-J)	Std. Error	Sig.[b]	95% Confidence Interval for Difference[b]	
					Lower Bound	Upper Bound
,0000	30,0000	-,302*	,014	,000	-,330	-,275
30,0000	,0000	,302*	,014	,000	,275	,330

Based on estimated marginal means

*. The mean difference is significant at the ,05 level.

b. Adjustment for multiple comparisons: Least Significant Difference (equivalent to no adjustments).

data

Tukey HSD[a,b]

Nahco3	N	Subset		
		1	2	3
1,00	12	,4723		
2,00	12		,5552	

3,00	12		,5577	
4,00	12			,6725
Sig.		1,000	,999	1,000

Means for groups in homogeneous subsets are displayed.
Based on observed means.
The error term is Mean Square(Error) = ,002.
a. Uses Harmonic Mean Sample Size = 12,000.
b. Alpha = ,05.

Chlorophyll b

Tests of Between-Subjects Effects

Dependent Variable:

Source	Type III Sum of Squares	df	Mean Square	F	Sig.	Partial Eta Squared
Corrected Model	1,165[a]	7	,166	59,504	,000	,912
Intercept	12,576	1	12,576	4495,419	,000	,991
Nahco3	,068	3	,023	8,114	,000	,378
salinity	,850	1	,850	303,755	,000	,884
Nahco3 * salinity	,247	3	,082	29,478	,000	,689
Error	,112	40	,003			
Total	13,853	48				
Corrected Total	1,277	47				

a. R Squared = ,912 (Adjusted R Squared = ,897)

Pairwise Comparisons

Dependent Variable:

(I) salinity		Mean Difference (I-J)	Std. Error	Sig.[b]	95% Confidence Interval for Difference[b]	
					Lower Bound	Upper Bound
,0000	30,0000	-,266*	,015	,000	-,297	-,235
30,0000	,0000	,266*	,015	,000	,235	,297

Based on estimated marginal means

*. The mean difference is significant at the ,05 level.

b. Adjustment for multiple comparisons: Least Significant Difference (equivalent to no adjustments).

Tukey HSD[a,b]

Nahco3	N	Subset	
		1	2
1,00	12	,4563	
2,00	12	,5065	,5065
3,00	12		,5235
4,00	12		,5611
Sig.		,109	,071

Means for groups in homogeneous subsets are displayed.
Based on observed means.
The error term is Mean Square(Error) = ,003.
a. Uses Harmonic Mean Sample Size = 12,000.
b. Alpha = ,05.
